# Line Immunoassay for Confirmation and Discrimination of Human T-Cell Lymphotropic Virus Infections in Inconclusive Western Blot Serum Samples from Brazil

**DOI:** 10.1128/JCM.01384-19

**Published:** 2019-12-23

**Authors:** Karoline R. Campos, Fred L. N. Santos, Vanessa da Silva Brito, Noilson L. S. Gonçalves, Thessika H. A. Araujo, Bernardo Galvão-Castro, Adele Caterino-de-Araujo

**Affiliations:** a Immunology Department, Adolfo Lutz Institute (IAL), São Paulo, São Paulo, Brazil; b Advanced Laboratory of Public Health, Gonçalo Moniz Institute (IGM), FIOCRUZ-BA, Salvador, Bahia, Brazil; c Integrated and Multidisciplinary HTLV Center, Bahiana School of Medicine and Public Health (EBMSP), Salvador, Bahia, Brazil; Memorial Sloan Kettering Cancer Center

**Keywords:** diagnostic, HTLV, confirmatory serological tests, LIA, WB

## Abstract

Difficulties in confirming and discriminating human T-cell lymphotropic virus type 1 (HTLV-1) and HTLV-2 infections by serological Western blot (WB) assays (HTLV Blot 2.4; MP Biomedicals) have been reported in Brazil, mainly in HIV/AIDS patients, with a large number of WB-indeterminate and WB-positive but HTLV-untypeable results. Nonetheless, a line immunoassay (LIA) (INNO-LIA HTLV-I/II; Fujirebio) provided enhanced specificity and sensitivity for confirming HTLV-1/2 infections. To add information concerning the improved ability of the LIA in relation to WB when applied to samples of individuals from different risk groups from Brazil, we performed the present study. Three groups were analyzed: group 1 (G1), with 62 samples from HIV/AIDS patients from São Paulo, SP (48 WB indeterminate and 14 HTLV untypeable); group 2 (G2), with 24 samples from patients with hepatitis B or hepatitis C from São Paulo (21 WB indeterminate and 3 HTLV untypeable; 17 HIV seropositive); and group 3 (G3), with 25 samples from an HTLV outpatient clinic in Salvador, Bahia (16 WB indeterminate and 9 HTLV untypeable; all HIV seronegative). Overall, the LIA confirmed HTLV-1/2 infection (HTLV-1, HTLV-2, or HTLV) in 66.1% (G1), 83.3% (G2), and 76.0% (G3) of samples. Interestingly, the majority of WB-indeterminate results were confirmed by the LIA as being HTLV-2 positive in G1 and G2 but not in G3, in which the samples were defined as being HTLV-1 or HTLV positive. These results agree with the virus types that circulate in such patients of different regions in Brazil and emphasize that the LIA is the best serological test for confirming HTLV-1 and HTLV-2 infections, independently of being applied in HTLV-monoinfected or HTLV-coinfected individuals.

## Abstract (precis)

Difficulties in confirming and discriminating human T-cell lymphotropic virus type 1 (HTLV-1) and HTLV-2 infections by serological Western blot (WB) assays (HTLV Blot 2.4; MP Biomedicals) have been reported in Brazil, mainly in HIV/AIDS patients, with a large number of WB-indeterminate and WB-positive but HTLV-untypeable results. Nonetheless, a line immunoassay (LIA) (INNO-LIA HTLV-I/II; Fujirebio) provided enhanced specificity and sensitivity for confirming HTLV-1/2 infections.

## INTRODUCTION

Despite regional variations, Brazil is one of the largest areas of the world where human T-cell lymphotropic virus type 1 (HTLV-1) is endemic (1). Higher rates of HTLV-1 infection have been detected in general populations in the north and northeast regions, while human T-cell lymphotropic virus type 2 (HTLV-2) is endemic among indigenous communities in the north as well as in injectable drug users in urban areas, mostly in southeastern in Brazil (2).

Difficulties in confirming the diagnosis of HTLV-1 and HTLV-2 by serological assays (Western blot [WB]) (HTLV Blot 2.3 and 2.4) have been reported in Brazil, due to a large number of WB-indeterminate and HTLV-positive but untypeable results, mainly in patients truly infected with HTLV-2 and/or HIV (3–5). Consequently, molecular assays have been employed to detect proviral DNA segments of HTLV-1 and HTLV-2 (*pol*, long terminal repeat [LTR], *env*, and *tax*) in peripheral blood mononuclear cells using nested PCR, PCR hybridization, and/or PCR-restriction fragment length polymorphism (RFLP) assays (6–11). Nonetheless, there was no consensus regarding the criteria to consider a blood sample truly infected by HTLV-1/2 using these techniques if positive for one or at least two DNA proviral segments of HTLV-1/2 (6, 10, 11). Subsequently, another molecular assay, real-time PCR or quantitative PCR (qPCR), was proposed as a confirmatory HTLV-1/2 molecular assay; however, low sensitivity was found when applied on HIV blood samples and on those from Brazilian patients infected with HTLV-2, which could be due to low HTLV-2 proviral loads (12–15).

In 1998, a new HTLV-1/2 serological confirmatory assay employed a line immunoassay (LIA) with a nylon membrane sensitized with the most relevant antigens, recombinant proteins, or synthetic peptides of HTLV-1 and HTLV-2 (16). This new immunoassay (INNO-LIA HTLV) demonstrated better results than WB, with improved sensitivity for the confirmation of HTLV-1 and HTLV-2 infections, thereby reducing numbers of WB-indeterminate results (16). However, the high cost of INNO-LIA prevented its routine adoption in Brazil. Consequently, few studies have compared the performances of LIA and WB in Brazil, one employing blood bank samples (17) and two using serum samples from HIV/AIDS patients (14, 15). These studies indicated that the LIA was the best assay to confirm or rule out HTLV-1/2 infection.

In Brazil, molecular biology laboratories are not widely available due to differences in socioeconomic conditions. Thus, the use of a confirmatory serological assay with high performance is essential and necessary for HTLV-1/2 diagnosis. Accordingly, the present study aimed to evaluate the use of INNO-LIA for clarifying WB-indeterminate and WB-HTLV-untypeable serum samples.

## MATERIALS AND METHODS

### Samples.

The serum samples employed in the present study were obtained from the biorepositories of the HTLV Research Laboratory (LPHTLV), Department of Immunology, Adolfo Lutz Institute (IAL), located in São Paulo, Brazil, and the Integrated and Multidisciplinary HTLV Center (CHTLV), located at the Bahiana School of Medicine and Public Health (EBMSP) in Salvador, Bahia, Brazil. Briefly, the samples from São Paulo were collected between 2012 and 2016 in the course of previous studies designed to detect the prevalence of HTLV-1/2 in HIV-infected individuals as well as in patients with hepatitis B virus (HBV) or hepatitis C virus (HCV) infection in the state of São Paulo, Brazil (14, 15, 18–20). The samples from Salvador, Bahia, were obtained by routine diagnostic procedures at an outpatient clinic in Salvador (CHTLV) from 2015 to 2017; these samples were additionally used to assess the performances of four commercially available HTLV serological screening tests in Brazil (21). Table 1 lists the characteristics of the samples collected from these two biorepositories (number of samples per sex and age) as well as HTLV-1/2 screening results and confirmatory WB assay results (HTLV Blot 2.4; MP Biomedicals). Only samples that were WB inconclusive (WB indeterminate or HTLV untypeable) were selected for analysis in the present investigation.

**TABLE 1:** Characteristics of the study groups whose serum samples were analyzed for the presence of HTLV-1/2 antibodies and results of HTLV-1/2 screening and Western blot confirmatory assays

Group^*a*^	Study population	No. of individuals	No. of samples	% of samples	Age (yr)		No. of screen-positive samples^*b*^	No. of WB-positive samples		No. of WB-inconclusive samples		% WB-inconclusive samples^*c*^	
					Mean	Min–max		HTLV-1	HTLV-2	HTLV	IND	HTLV	IND
G1	HIV	4,395					311	97	65	16	66	5.14	21.22
Male			2,935	66.78	39.12	16–84							
Female			1,459	33.20	40.36	16–83							

Total			4,395	100	39.74	16–84							


G2	HBV/HCV	3,228					99	45	29	3	21	3.03	21.21
Male			1,749	54.18	48.40	14–88							
Female			1,479	45.82	48.50	13–94							

Total			3,228	100	48.70	13–94							


G3^*d*^	Monoinfection	362					207	127^*e*^	36^*e*^	11	27	5.31	13.04
Male			105	29.01	39.00	24–60							
Female			257	70.99	42.20	16–73							

Total			362	100	41.04	16–73							

a G1, group of HIV/AIDS patients from São Paulo, SP, Brazil; G2, group of patients with hepatitis B virus (HBV) and hepatitis C virus (HCV) from São Paulo, SP; G3, group of individuals from HTLV outpatient clinics in Salvador, BA, Brazil.

b Screening assays as described in Materials and Methods.

c Western blot results according to the manufacturer’s criteria for HTLV Blot 2.4 (MP Biomedicals). IND, indeterminate.

d Age data obtained only from patients with WB-inconclusive results.

e Five samples were positive for both HTLV-1 and HTLV-2.

Of the 145 samples with WB-inconclusive results, a volume sufficient for immunoassaying was present in 111; these samples were used to determine the ability of the LIA (INNO-LIA HTLV; Fujirebio) to confirm and discriminate HTLV-1/2-specific antibodies. The evaluated samples originated from three groups of patients: group 1 (G1), with 62 samples (48 WB indeterminate and 14 HTLV untypeable) obtained in the course of routine HTLV diagnosis at the Adolfo Lutz Institute or from the São Paulo Sexually Transmitted Disease/AIDS Reference and Training Center (CRT DST/AIDS-São Paulo), all of which were obtained from individuals who were known to be HIV seropositive; group 2 (G2), with 24 samples (21 WB indeterminate and 3 HTLV untypeable) that were collected from patients who were initially seen at gastroenterology centers in São Paulo, 14 of whom had HBV and 10 of whom had HCV infection, with 17 of these being HIV seropositive (10 with HBV and 7 with HCV); and group 3 (G3), with 25 HTLV-monoinfected samples from patients seen at the CHTLV outpatient clinic in Salvador, Bahia (16 WB indeterminate and 9 HTLV untypeable).

### Screening assays.

The samples from São Paulo were screened for the presence of HTLV-1/2 antibodies by two enzyme immunoassays (EIAs): Murex HTLV-I+II (DiaSorin S.p.A., Dartford, UK) and Gold ELISA (enzyme-linked immunosorbent assay) HTLV-1/2 (REM Indústria e Comércio Ltd., São Paulo, Brazil). The samples from Salvador were initially screened by the Ortho HTLV-1/HTLV-2 Ab-Capture ELISA system (Ortho Clinical Diagnostics, Raritan, NJ, USA) as well as by four other HTLV-1/2 screening tests commercially available in Brazil: three EIAs (Murex HTLV-I+II [DiaSorin S.p.A., Dartford, UK], anti-HTLV-1/2 Sym solution [Symbiosis Diagnóstica Ltd., Leme, Brazil], and Gold ELISA HTLV-1/2 [REM Indústria e Comércio Ltd., São Paulo, Brazil]) and one chemiluminescence assay (CLIA) kit (Architect rHTLV-1/2; Abbott Diagnostics Division, Wiesbaden, Germany). All assays were performed according to the manufacturers’ instructions, which were also used to interpret results. Cutoff values and gray zones were calculated for each assay, and samples considered reactive or inconclusive in screening were submitted for a confirmatory assay.

### Confirmatory assays.

A WB assay (HTLV Blot 2.4; MP Biomedicals Asia Pacific Pte. Ltd., Singapore) was used to confirm HTLV-1 and HTLV-2 infection for all the previous studies that generated the samples analyzed here, and these results were interpreted according to the stringent criteria provided by the manufacturer. Briefly, HTLV-1-positive serum samples were defined as having the presence of Gag (p19 with or without p24) and two Env (GD21 and rgp46-I) bands. HTLV-2-positive samples were defined as demonstrating reactivity to Gag (p24 with or without p19) and two Env (GD21 and rgp46-II) bands. Samples that showed the presence of antibodies to both Gag (p19 and p24) and Env (GD21) were defined as being HTLV positive but were considered untypeable. Any other pattern of bands was deemed to be indeterminate.

The present study employed an LIA (INNO-LIA HTLV I/II; Fujirebio, Europe NV, Belgium) in an attempt to confirm and/or discriminate samples with inconclusive results by WB (i.e., WB indeterminate or HTLV positive but untypeable). The strips used in the LIA contain antigens for validation, confirmation, and discrimination. For validation, the line marked by each sample was compared to the control line, and a score ranging from +/− to +3 was assigned. The confirmatory antigens included Gag p19-I/II, Gag p24-I/II, Env gp46-I/II, and Env gp21-I/II. No bands or the occurrence of a single band (Gag p19-I/II, Gag p24-I/II, or Env gp46-I/II) denoted a negative result. The presence of one band (Env gp21-I/II) or two bands (except Env gp21-I/II) indicated indeterminate results, while two bands (Env gp21-I/II and Gag p19-I/II, Gag p24-I/II, or Env gp46-I/II) indicated HTLV positivity. Three discriminatory bands (Gag p19-I, Env gp46-I, and Env gp46-II) were considered as follows: HTLV-1 positivity was indicated by reactivity to Gag p19-I and/or Env gp46-I, while HTLV-2 positivity was found when samples showed Env gp46-II or a higher intensity of the Env gp46-II band than the Gag p19-I and Env gp46-I bands.

### Statistical analyses.

Differences in the numbers of males and females in each group were evaluated statistically using the chi-square test. GraphPad Prism software version 5.03 (GraphPad, San Diego, CA, USA) was used for age comparisons between groups using Kruskal-Wallis analysis of variance (ANOVA), complemented with Dunn’s multiple-comparison test. Results with a *P* value of ≤0.05 were considered statistically significant.

### Ethical considerations.

The present research protocol was approved by the Institutional Review Board of the IAL in São Paulo, Brazil (protocol no. 106D/2012, 62H/2015, and 21I/2016) and by the Institutional Research Board (IRB) of the EBMSP in Salvador, Bahia, Brazil (protocol no. 464.286). All procedures were performed in accordance with the principles established in the Declaration of Helsinki and its subsequent revisions.

## RESULTS

The characteristics of the patients (sex and age) and the distribution of WB-inconclusive results in each study group are presented in Table 2. More males were found among the HBV/HCV-infected patients in G2, with significant differences in relation to HIV/AIDS patients in G1 and HTLV patients in G3 (*P* = 0.0048). A comparative analysis of age among the groups showed no significant differences, although the individuals in G2 were older overall. Concerning the distribution of WB-inconclusive samples, the three groups contained more WB-indeterminate than HTLV-untypeable samples: 77.4% in G1, 87.5% in G2, and 64.0% in G3.

**TABLE 2:** Characteristics (sex and age) and numbers of individuals whose serum samples yielded HTLV-inconclusive WB results and were available for the LIA in each study group

Parameter	Value for group^*a*^		
	G1 (*n* = 62)	G2 (*n* = 24)	G3 (*n* = 25)
No. (%) of individuals of sex^*b*^			
Male	31 (50.00)	20 (83.30)	10 (40.00)
Female	31 (50.00)	4 (16.70)	15 (60.00)
Mean age (yr) (min–max)	44.06 (18–68)	49.50 (35–76)	41.08 (16–73)
No. of WB-indeterminate results^*c*^	48	21	16
No. of WB HTLV-untypeable results^*c*^	14	3	9

a G1, group of HIV/AIDS patients from São Paulo, SP, Brazil; G2, group of patients with HBV and HCV infection from São Paulo, SP; G3, group of individuals from an HTLV outpatient clinic in Salvador, BA, Brazil.

b The *P* value for sex was 0.0048, which was statistically significant using a chi-square test.

c Indeterminate and HTLV-untypeable results according to the manufacturer’s criteria for HTLV Blot 2.4 (MP Biomedicals).

**FIG 1:**
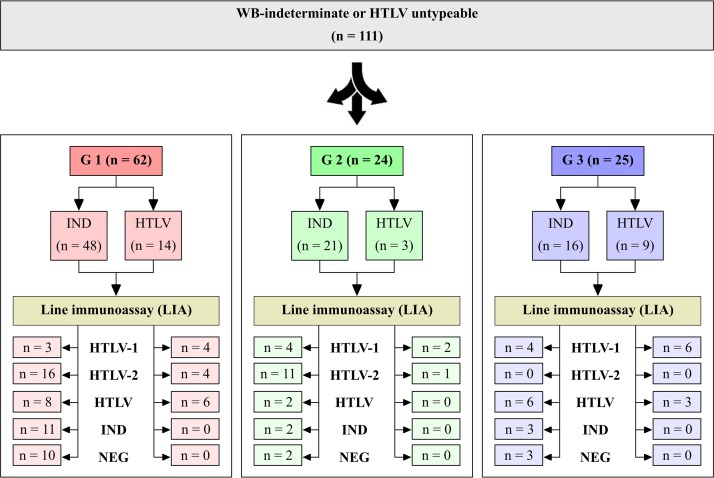
Numbers of serum samples in each study group that scored WB indeterminate or HTLV untypeable in previous analyses and HTLV infections confirmed or excluded by a Western blot (WB) assay (HTLV Blot 2.4; MP Biomedicals) and a line immunoassay (LIA) (INNO-LIA HTLV-I/II; Fujirebio). IND, indeterminate; NEG, negative.

The LIA provided confirmation of HTLV-1/2 infection (HTLV-1, HTLV-2, or HTLV) in 66.1% (G1), 83.3% (G2), and 76.0% (G3) of the samples analyzed. Interestingly, most WB-indeterminate results in G1 and G2 were confirmed to be HTLV-2 positive by the LIA, but this was not the case in G3. In G3, only HTLV-1 (40.0%)- and HTLV (36.0%)-positive samples were detected among both WB-indeterminate and HTLV-untypeable samples (Fig. 1).

Table 3 shows the WB-indeterminate profiles detected in the present study, the numbers of samples that presented each profile, and the numbers and percentages of samples with confirmed HTLV-1/2 infection. These results indicate that the WB patterns showing Env bands (GD21 and/or rgp46-I or -II) plus p19 or p24 were confirmed as being positive for HTLV-1/2 infection by the LIA. The overall patterns from the WB-inconclusive samples (*n* = 111) and the patterns returned by the LIA are presented in Table 4, revealing that the majority of indeterminate WB profiles that were not confirmed as being positive for HTLV-1/2 infection by the LIA presented only Gag bands in G1, only GD21 in G2, and one of three bands (GD21, rgp46-II, and p24) in G3. It is noteworthy that among 26 HTLV-positive but untypeable samples (14 in G1, 3 in G2, and 9 in G3), after the LIA, 28.6% were confirmed as being HTLV-1 positive, 28.6% were confirmed as being HTLV-2 positive, and 42.8% remained HTLV untypeable in G1. In G2, 66.7% were confirmed as being HTLV-1 positive and 33.3% were confirmed as being HTLV-2 positive, and in G3, 66.7% were confirmed as being HTLV-1 positive and 33.3% remained HTLV untypeable (Fig. 1 and Table 4).

**TABLE 3:** WB-indeterminate profiles detected in serum samples from each study group and numbers and percentages of serum samples with HTLV-1/2 infection confirmed by LIA

WB-indeterminate profile^*a*^	No. of samples with profile				No. of LIA-positive samples (%)^*b*^
	G1 (*n* = 48)	G2 (*n* = 21)	G3 (*n* = 16)	Total	
GD21, p24	10	3		13	12 (92.30)
rgp46 only (I or II)		5	1	6	5 (83.33)
GD21, p19	3	3	3	9	8 (88.89)
p24, rgp46-II	4	3		7	7 (100.00)
GD21, rgp46 (I and/or II)	7	1	4	12	9 (75.00)
GD21	4	4	6	14	3 (21.43)
p19	5			5	1 (20.00)
p24	4	1	1	6	1 (16.67)
GD21, p24, rgp46-I	3			3	1 (33.33)
p19, rgp46-I	1		1	2	2 (100.00)
GD21, gp21, rgp46-II	1			1	1 (100.00)
GD21, p19, p26	1			1	1 (100.00)
GD21, p24, p32, p36	1			1	1 (100.00)
p19, p24, rgp46-II		1		1	1 (100.00)
p24, p36, rgp46-II	1			1	1 (100.00)
p19, p24	1			1	
p19, p26, p28, p53	2			2	

a According to criteria established by the manufacturer of HTLV Blot 2.4 (MP Biomedicals).

b According to the requirements established by the manufacturer of INNO-LIA HTLV-I/II (Fujirebio).

**TABLE 4:** Final results and profiles obtained with confirmatory serological assays for detecting HTLV-1/2 antibodies in 111 serum samples that yielded WB-inconclusive results and were tested by LIA

Group and WB result for each sample^*a*^	WB profile	LIA result^*b*^	LIA profile	Final result
G1				
Indeterminate	GD21, p24	HTLV	p19-I/II, p24-I/II, gp21-I/II	HTLV
HTLV	GD21, p19, p24	HTLV	p19-I/II, p24-I/II, gp21-I/II	HTLV
HTLV	GD21, p19, p24, p26, p28, p32, p36, p53	HTLV	p19-I/II, gp21-I/II	HTLV
Indeterminate	p19, p26, p28, p53	Negative	p19-I/II	Negative
Indeterminate	GD21,^*c*^ p24^*c*^	HTLV	p19-I/II, p24-I/II, gp46-I/II, gp21-I/II	HTLV
Indeterminate	GD21, p24	HTLV	p19-I/II, p24-I/II, gp21-I/II	HTLV
Indeterminate	p24, rgp46-II	HTLV-2	p19-I/II, p24-I/II, gp46-I/II, gp46-II	HTLV-2
Indeterminate	GD21	Indeterminate	gp21-I/II	Indeterminate
Indeterminate	p24	Indeterminate	p19-I/II, p24-I/II	Indeterminate
Indeterminate	GD21, rgp46-II	HTLV-2	gp46-I/II, gp21-I/II, gp46-II	HTLV-2
Indeterminate	GD21, p19	HTLV-1	p19-I/II, gp21-I/II, p19-I	HTLV-1
Indeterminate	p24, p36, rgp46-II	HTLV-2	p19-I/II, p24-I/II, gp46-I/II, gp21-I/II, gp46-II	HTLV-2
Indeterminate	GD21	Indeterminate	gp21-I/II	Indeterminate
Indeterminate	p19, p26, p28, p53	Negative	p19-I/II	Negative
Indeterminate	p24^*c*^	Indeterminate	gp21-I/II	Indeterminate
HTLV	GD21,^*c*^ p19,^*c*^ p24	HTLV-1	p19-I/II, p24-I/II, gp21-I/II, p19-I	HTLV-1
Indeterminate	p19	Negative	p19-I/II, p19-I	Negative
Indeterminate	p19, p24	Negative	p24-I/II	Negative
Indeterminate	GD21,^*c*^ p24^*c*^	Indeterminate	gp21-I/II	Indeterminate
HTLV	GD21, p19, p24	HTLV-2	p19-I/II, p24-I/II, gp46-I/II, gp21-I/II, gp46-II	HTLV-2
Indeterminate	GD21, gp21, rgp46-II	HTLV-2	p19-I/II, gp46-I/II, gp21-I/II, gp46-II	HTLV-2
Indeterminate	GD21, p24	HTLV	p19-I/II, p24-I/II, gp46,-I/II, gp21-I/II	HTLV
Indeterminate	GD21, rgp46-II, rgp46-I	Negative	No bands	Negative
Indeterminate	GD21, p24,^*c*^ rgp46-I	HTLV-1	p19-I/II, gp46-I/II, gp21-I/II, gp46-I	HTLV-1
Indeterminate	GD21^*c*^	Negative	p19-I/II	Negative
HTLV	GD21, p19, p24, p26, p28, p32	HTLV	p19-I/II, p24-I/II, gp21-I/II	HTLV
HTLV	GD21, p19, p24	HTLV	p19-I/II, gp21-I/II	HTLV
Indeterminate	p19,^*c*^ rgp46-I	HTLV-1	p19-I/II, p24-I/II, gp46-I/II, gp21-I/II, gp46-I	HTLV-1
Indeterminate	p24,^*c*^ rgp46-II	HTLV-2	gp46-I/II, gp21-I/II, gp46-II	HTLV-2
HTLV	GD21, p19, p24, p26, p32, p36, p53	HTLV	p19-I/II, p24-I/II, gp46-I/II, gp21-I/II	HTLV
HTLV	GD21, p19, p24	HTLV-2	p19-I/II, p24-I/II, gp46-I/II, gp21-I/II, gp46-II	HTLV-2
Indeterminate	GD21, p24	HTLV-2	gp46-I/II, gp21-I/II, gp46-II	HTLV-2
Indeterminate	GD21, p19	HTLV	p19-I/II, gp46-I/II, gp21-I/II	HTLV
HTLV	GD21, p19, p24	HTLV	p19-I/II, p24-I/II, gp21-I/II	HTLV
HTLV	GD21, p19, p24	HTLV-2	p19-I/II, p24-I/II, gp46-I/II, gp21-I/II, gp46-II	HTLV-2
Indeterminate	GD21,^*c*^ p24	HTLV-2	gp46-I/II, gp21-I/II, gp46-II	HTLV-2
Indeterminate	GD21, p24, p32, p36	HTLV-2	p19-I/II, p24-I/II, gp46-I/II, gp21-I/II, gp46-II	HTLV-2
Indeterminate	p24,^*c*^ rgp46-II	HTLV-2	gp46-I/II, gp21-I/II, gp46-II	HTLV-2
Indeterminate	p24, rgp46-II	HTLV-2	gp46-I/II, gp21-I/II, gp46-II	HTLV-2
Indeterminate	GD21, p24	HTLV	p19-I/II, p24-I/II, gp21-I/II	HTLV
Indeterminate	p24^*c*^	Negative	No bands	Negative
Indeterminate	GD21	Indeterminate	gp21-I/II	Indeterminate
Indeterminate	GD21, rgp46-II	HTLV-2	gp46-I/II, gp21-I/II, gp46II	HTLV-2
Indeterminate	p19	Negative	p19-I/II	Negative
Indeterminate	GD21, p24,^*c*^ rgp46-I^*c*^	Indeterminate	gp21-I/II	Indeterminate
Indeterminate	GD21, p19, p26	HTLV	p19-I/II, gp46-I/II, gp21-I/II	HTLV
Indeterminate	GD21, rgp46-II	Indeterminate	gp21-I/II	Indeterminate
Indeterminate	GD21, p24,^*c*^ rgp46-I^*c*^	Indeterminate	gp21-I/II	Indeterminate
Indeterminate	GD21, rgp46-I	Indeterminate	gp21-I/II	Indeterminate
HTLV	GD21, p19, p24	HTLV-2	p19-I/II, p24-I/II, gp21-I/II, gp46-II	HTLV-2
Indeterminate	GD21, rgp46-II, rgp46-I^*c*^	HTLV-2	gp46-I/II, gp21-I/II, gp46-II	HTLV-2
Indeterminate	GD21, p24	HTLV-2	p19-I/II, p24-I/II, gp46-I/II, gp21-I/II, gp46-II	HTLV-2
Indeterminate	p24	Indeterminate	p19-I/II, p24-I/II	Indeterminate
Indeterminate	p19	Negative	p19-I/II	Negative
Indeterminate	p19^*c*^	HTLV	p19-I/II, gp21-I/II	HTLV
Indeterminate	GD21, rgp46-II	HTLV-2	gp46-I/II, gp21-I/II, gp46-II	HTLV-2
Indeterminate	GD21,^*c*^ p19	HTLV-2	p19-I/II, gp46-I/II, gp21-I/II, gp46-II	HTLV-2
HTLV	GD21, p19, p24, p26, p32, p36	HTLV-1	p19-I/II, p24-I/II, gp46-I/II, gp21-I/II, p19-I, gp46-I	HTLV-1
Indeterminate	GD21, p24	HTLV-2	p19-I/II, gp46-I/II, gp21-I/II, gp46-II	HTLV-2
HTLV	GD21, p19, p24	HTLV-1	p19-I/II, p24-I/II, gp21-I/II, p19-I	HTLV-1
Indeterminate	p19	Negative	p19-I/II	Negative
HTLV	GD21, p19, p24	HTLV-1	p19-I/II, p24-I/II, gp21-I/II, p19-I	HTLV-1
G2				
Indeterminate	rgp46-I	HTLV-1	p24-I/II, gp46-I/II, gp21-I/II, gp46-I	HTLV-1
Indeterminate	p24, rgp46-II	HTLV-2	p24-I/II, gp46-I/II, gp21-I/II, gp46-I,^*c*^ gp46-II	HTLV-2
HTLV	GD21, p19, p24, p26,^*c*^ p28,^*c*^ p32,^*c*^ p36^*c*^	HTLV-1	p19-I/II, p24-I/II, gp46-I/II, gp21-I/II, p19-I, gp46-I	HTLV-1
Indeterminate	GD21^*c*^	Negative	No bands	Negative
Indeterminate	rgp46-I^*c*^	HTLV-1	gp46-I/II, gp21-I/II, gp46-I	HTLV-1
Indeterminate	GD21,^*c*^ rgp46-I	HTLV-1	gp46-I/II, gp21-I/II, gp46-I	HTLV-1
Indeterminate	GD21, p19^*c*^	Indeterminate	gp21-I/II	Indeterminate
Indeterminate	p24, rgp46-II	HTLV-2	gp46-I/II, gp21-I/II, gp46-II	HTLV-2
Indeterminate	rgp46-II	HTLV-2	p19-I/II, gp46-I/II, gp21-I/II, gp46-II	HTLV-2
Indeterminate	GD21^*c*^	HTLV	p19-I/II, gp21-I/II	HTLV
HTLV	GD21, p19, p24	HTLV-1	p19-I/II, p24-I/II, gp46-I/II, gp21-I/II, gp46-I	HTLV-1
Indeterminate	GD21	Negative	No bands	Negative
Indeterminate	GD21, p19	HTLV	p19-I/II, p24-I/II, gp21-I/II	HTLV
Indeterminate	p24, rgp46-II	HTLV-2	p19-I/II, p24-I/II, gp46-I/II, gp21-I/II, gp46-II	HTLV-2
Indeterminate	GD21^*c*^	Indeterminate	gp21-I/II	Indeterminate
Indeterminate	p24	HTLV-2	gp46-I/II, gp21-I/II, gp46II^*c*^	HTLV-2
Indeterminate	GD21, p24	HTLV-2	p19-I/II, gp46-I/II, gp21-I/II, gp46-II	HTLV-2
Indeterminate	p19, p24, rgp46-II	HTLV-2	p19-I/II, p24-I/II, gp46-I/II, gp21-I/II, gp46-II	HTLV-2
Indeterminate	GD21, p24	HTLV-2	p19-I/II, p24-I/II, gp46-I/II, gp21-I/II, gp46-II	HTLV-2
Indeterminate	rgp46-II	HTLV-2	p19-I/II, gp46-I/II, gp21-I/II, gp46-II	HTLV-2
Indeterminate	rgp46-II	HTLV-2	p24-I/II, gp46-I/II, gp21-I/II, gp46-II	HTLV-2
Indeterminate	GD21, p24	HTLV-2	p19-I/II, p24-I/II, gp46-I/II, gp21-I/II, gp46-II	HTLV-2
Indeterminate	GD21,^*c*^ p19^*c*^	HTLV-1	p19-I/II, p24-I/II, gp46-I/II, gp21-I/II, p19-I,^*c*^ gp46-I	HTLV-1
HTLV	GD21,^*c*^ p19,^*c*^ p24	HTLV-2	p19-I/II, p24-I/II, gp46-I/II, gp21-I/II, gp46-II^*c*^	HTLV-2

G3				
HTLV	GD21, p19, p24, p26,^*c*^ p28,^*c*^ p36^*c*^	HTLV-1	p19-I/II, p24-I/II, gp46-I/II, gp21-I/II, p19-I, gp46-I	HTLV-1
HTLV	GD21, p19, p24, p26,^*c*^ p28,^*c*^ p32,^*c*^ p36,^*c*^ gp46^*c*^	HTLV-1	p19-I/II, p24-I/II, gp46-I/II, gp21-I/II, gp46-I	HTLV-1
HTLV	GD21,^*c*^ p19, p24^*c*^	HTLV	p19-I/II, p24-I/II, gp46-I/II, gp21-I/II	HTLV
HTLV	GD21, p19, p24, p26c, p28c, p36, gp46	HTLV	p19-I/II, p24-I/II, gp46-I/II, gp21-I/II	HTLV
HTLV	GD21, p19, p24, p26, p28, p32, p36, gp46, p53	HTLV-1	p19-I/II, p24-I/II, gp46-I/II, gp21-I/II, p19-I	HTLV-1
HTLV	GD21, p19, p24, p26, p28, p32, p36	HTLV-1	p19-I/II, p24-I/II, gp46-I/II, gp21-I/II, p19-I	HTLV-1
Indeterminate	GD21,^*c*^ p19^*c*^	HTLV	p19-I/II, gp46-I/II, gp21-I/II	HTLV
HTLV	GD21, p19, p24^*c*^	HTLV-1	p19-I/II, p24-I/II, gp46-I/II, gp21-I/II, p19-I	HTLV-1
Indeterminate	GD21, rgp46-II	HTLV	p19-I/II, gp21-I/II	HTLV
HTLV	GD21, p19, p24,^*c*^ p26, p28, p32, p36	HTLV-1	p19-I/II, p24-I/II, gp46-I/II, gp21-I/II, p19-I, gp46-I	HTLV-1
Indeterminate	GD21, rgp46-I	HTLV-1	p19-I/II, gp46-I/II, gp21-I/II, gp46-I	HTLV-1
Indeterminate	GD21, p19	HTLV	p19-I/II, gp21-I/II	HTLV
Indeterminate	GD21^*c*^	Indeterminate	gp21-I/II	Indeterminate
Indeterminate	GD21	Indeterminate	gp21-I/II	Indeterminate
Indeterminate	GD21	HTLV-1	gp46-I/II, gp21-I/II, gp46-I	HTLV-1
Indeterminate	GD21	Negative	No bands	Negative
Indeterminate	p24^*c*^	Negative	No bands	Negative
Indeterminate	rgp46-II^*c*^	Negative	No bands	Negative
Indeterminate	GD21, rgp46-I	HTLV-1	p19-I/II, gp46-I/II, gp21-I/II, gp46-I	HTLV-1
HTLV	GD21, p19, p24	HTLV	p19-I/II, gp21-I/II	HTLV
Indeterminate	GD21	HTLV	p19-I/II, gp21-I/II	HTLV
Indeterminate	GD21, rgp46-II^*c*^	HTLV	p19-I/II, gp21-I/II	HTLV
Indeterminate	GD21	Indeterminate	gp21-I/II	Indeterminate
Indeterminate	GD21, p19	HTLV	p19-I/II, gp46-I/II, gp21-I/II	HTLV
Indeterminate	p19, rgp46-I	HTLV-1	p19-I/II, gp46-I/II, gp21-I/II, gp46-I	HTLV-1

a WB, Western blot (HTLV Blot 2.4; MP Biomedicals).

b LIA, line immunoassay (INNO-LIA HTLV-I/II; Fujirebio).

c Faint bands.

## DISCUSSION

HTLV-1- and HTLV-2-seroindeterminate WB results are prevalent worldwide, with rates fluctuating according to country and study group (geographic areas and populations where the disease is or is not endemic). Several attempts have been made to improve WB sensitivity and specificity, such as adding HTLV-1 and HTLV-2 recombinant envelope proteins and transmembrane protein to the HTLV-1 viral lysate. These include rgp46-I, rgp46-II, and GD21, the latter of which blocks the cross-reactivity of gp21 with Plasmodium falciparum in regions where malaria is endemic (22, 23). In spite of these efforts, Blot 2.4 continues to yield high rates of WB-indeterminate and/or untypeable HTLV results (4–8, 10, 11, 13–17, 21). The present study found similar results and disclosed that in populations presenting a high risk of acquiring viral infections (G1 and G2) as well in the general population (G3) of Brazil, a large number of WB-inconclusive results were detected. Several hypotheses were taken into consideration for these WB-inconclusive results, such as low HTLV-1 and HTLV-2 proviral loads, mutations in the provirus (defective particles), low-level production of viral antigens consequently leading to a low level of specific antibody production, the seroconversion period, cross-reactivity with other antigens or viruses, coinfection with HIV, and the use of antiretroviral therapy, among others (5, 12–15, 24–27).

Interestingly, the majority of WB-indeterminate results that were not confirmed by seroconversion or PCR assays were due to cross-reactivity with Gag antigens. For instance, the WB-indeterminate pattern exhibiting p19, p26, p28, p32, p36, and p53, termed the HTLV-1 Gag indeterminate profile (HGIP), has been detected in epidemiological studies, mostly in Cameroon and in the Caribbean, but was not associated with true HTLV-1 infection (23, 24). This pattern was not frequently described in Brazilian WB-indeterminate samples (5, 13, 15, 26) and was also not observed in any samples analyzed in the present study. Although two samples here presented an incomplete HGIP pattern (without p32 and p36 bands), only the p19-I/II band was detected by the LIA. Thus, these samples were considered HTLV negative according to the manufacturer’s criteria for the LIA. Corroborating the LIA result, the same profile (only the p19-I/II band) was detected when we analyzed samples from five patients who attended HTLV outpatient clinics in São Paulo during the years 2000 to 2006, which exhibited p19, p26, p28, and p36 WB bands (5).

Moreover, we conducted a retrospective analysis of 108 well-characterized blood samples from patients from São Paulo (tested by two serological assays [WB and LIA] and two molecular assays [qPCR for *pol* and PCR-RFLP analysis for *tax*]) and confirmed a series of more than 10 HTLV-1 and 10 HTLV-2 samples as being positive by both WB and LIA criteria, and they were concordant. In addition, we revised data for two samples that were HTLV-1 and -2 positive by WB, one of which was confirmed to be HTLV-1 and HTLV-2 positive by the LIA and molecular assays (15). Additionally, we tested two other HTLV-1 and -2 WB-positive samples by the LIA, and only HTLV-1 was confirmed, taking into account the intensity of the discriminatory bands (p19-I, gp46-I, and gp46-II). Of note, the WB-indeterminate profile (strong GD21 and p28 bands) described in Pygmies and Bantus living in the southern Cameroonian rainforest (28) has not been detected in any study conducted in São Paulo (5, 13, 15) and here.

Although PCR assays presented lower sensitivity than WB in detecting true HTLV-1/2 infection in HIV-HTLV-coinfected individuals in São Paulo, Brazil, the molecular assays were able to confirm and discriminate between HTLV-1 and HTLV-2 in some WB-indeterminate and HTLV-untypeable cases, indicating that both serological and molecular assays are useful for HTLV diagnosis (13, 15). Due to the presence of large numbers of WB-indeterminate samples, coupled with the high cost of obtaining WB assays and LIAs in Brazil, we recently proposed an algorithm that employs qPCR to confirm HTLV infection, followed by testing any PCR-negative samples by WB or an LIA. This strategy was shown to reduce costs and improve the diagnostic accuracy of HTLV-1/2 detection (13, 15). Nonetheless, due to highly divergent socioeconomic conditions among different regions in Brazil, in laboratories without the means to perform molecular assays, high-performance serological testing presents an acceptable alternative.

Some studies of HTLV diagnosis conducted in blood donors in Latin America (considered an area where HTLV-1/2 is endemic) have reported differing numbers of WB-indeterminate samples, which were subsequently confirmed to be positive by PCR (29–31). Also, in blood donors from another area where the disease is endemic, northeast Iran, WB-indeterminate samples were found to be positive by PCR, and the most prevalent WB bands presented various combinations of rgp46-I, GD21, and gp21 (32).

In corroboration with these findings, the majority of serum samples here that presented WB patterns of GD21 and/or rgp46-I or -II plus p19 or p24 were subsequently confirmed to be positive by the LIA. These types of WB patterns were observed in G1, and PCR assays demonstrated HTLV-1 or HTLV-2 positivity (data not shown) (15). In addition, the majority of serum samples that presented only Gag bands in the WB analysis were negative for HTLV-1/2 infection by the LIA. Of note, one blood sample from G1 that showed a faint GD21 band in the WB analysis tested negative for HTLV-1/2 by both the LIA and PCR. Another serum sample presenting a GD21, rgp46-I, and rgp46-II WB pattern was found to be negative by the LIA; unfortunately, this sample could not be analyzed by PCR because only serum was sent to the laboratory for analysis. However, retesting of this serum sample by WB and the LIA confirmed the discrepant results. It is interesting to note that in serum samples (*n* = 14) that tested HTLV untypeable by the LIA, PCR confirmed the presence of HTLV-1 in 5 samples and HTLV-2 in another 2 samples from G1 (data not shown), emphasizing the need for employing molecular assays to confirm an HTLV diagnosis in patients with HIV-HTLV coinfection.

In G2, the majority of WB-indeterminate patterns presented either GD21 alone or this protein in association with one Gag or envelope band. The LIA confirmed the presence of HTLV-2 in 11/21 (52.4%) of the WB-indeterminate samples. The high number of HTLV-2-positive samples in G2 leads us to suppose that these patients acquired HBV and HCV, as well HTLV-1/2 and HIV, at the same time, probably by the parenteral route and prior to the time when serological testing for HIV and HBV (1989), and subsequently for HTLV and HCV (1993), became mandatory in blood banks throughout Brazil; in addition, intravenous drug addiction was more frequent in this country, as previously described (18–20). Corroborating this hypothesis, older age and male sex predominated in G2. Regarding the lack of WB detection of truly HTLV-2-infected samples, we have described this difficulty since 2000 (8, 13, 15) and hypothesized that the rgp46-II (K55) present in the WB strip is not as sensitive for the detection antibodies to the HTLV-2 strains that circulate in Brazil (HTLV-2a subtype, variant 2c) (33). This seems not to be the case for the gp46-II present in the LIA strip.

Concerning the two WB-indeterminate samples in G2 with negative HTLV results by the LIA, both presented reactivity for GD21 in the WB analysis, and one of the samples showed a faint band. Curiously, the LIA demonstrates the best performance in this group of patients, with 20/24 (83.3%) of HTLV-positive samples being detected among the WB-inconclusive samples. Unfortunately, only plasma/serum samples from these patients were available for analysis, which did not allow the use of PCR to perform a comparative analysis of serological and molecular results. Nonetheless, associations between HTLV-1/2 and hepatitis B and C have been reported in several studies conducted in Brazil and elsewhere (18–20, 24, 29, 34).

The WB-inconclusive patterns in G3 were quite different from those in the other groups analyzed. Several of the HTLV-untypeable samples demonstrated the presence of almost all bands corresponding to the HTLV-1 viral lysate, without reactivity to rgp46-I, and six of nine were subsequently confirmed to be HTLV-1 positive by the LIA. Twelve out of the 25 WB-inconclusive samples that could be submitted for PCR (9 WB indeterminate and 3 HTLV indeterminate), 11 were confirmed to be HTLV-1 infected, 6 of which were HTLV untypeable by LIAs (data not shown). In addition, the serum samples that tested negative by LIAs presented three different WB-indeterminate patterns: (i) GD21, (ii) p24 (faint band), and (iii) rgp46-II (faint band). Only one of these samples could be tested by PCR and presented an HTLV-negative result (data not shown). In summary, the samples in G3 were confirmed to be positive for HTLV-1 or HTLV but not HTLV-2 infection. This finding could be partially related to the ethnic origin of the included individuals (African descendants), the lack of HIV infection in this group, and the characteristics of the patients seen at HTLV outpatient clinics in Salvador, BA (35).

Of note, the reasons described above to explain WB-inconclusive results could also be applied to the PCR-negative results in truly HTLV-1/2-infected individuals, including the low proviral loads in HIV/AIDS patients undergoing antiretroviral therapy in G1 and, in some cases, in G2 (13–15); the observation that HTLV-2 infection is known to show low proviral loads (11, 12, 25); the presence of defective provirus not detected by the primers employed in the PCR assays (27); and infection with other viruses, such as HTLV-3 or HTLV-4, which can be detected only by using specific primers (36, 37).

It is noteworthy that since the discovery of HTLV-3 and HTLV-4 in central Africa (36, 37), studies have been conducted to ascertain whether assays that are commercially available and employed for HTLV-1/2 diagnosis are able to detect these new HTLVs (38–40). The results obtained confirmed that HTLV-1/2 screening assays are sensitive for the detection of antibodies to HTLV-3 and HTLV-4 (38) and disclosed discordant and misclassified results in relation to confirmatory serological assays (WB and LIA) (36–41).

In fact, we could not rule out HTLV-3 and HTLV-4 infections in Brazil, since populations in central Africa migrated from Africa and Australia to the American continent before Asiatic population migration, and their descendants, such as the Amerindians, could maintain such viruses or spread them to the general population, which could justify the frequent presence of WB-indeterminate results in Amerindians, as previously described (42).

In relation to the LIA, although it presented the best performance in detecting HTLV-1 and HTLV-2, we could not exclude misclassified positive results, as occurred in HTLV-3- and HTLV-4-infected individuals, who could be erroneously diagnosed as being infected with HTLV-2 (36–38).

Of note, despite the fact that the LIA demonstrated better performance than WB in the serological diagnosis of both HTLV-1 and HTLV-2, additional considerations are warranted for both assays. With respect to WB, we consider the lack of an ability to score the intensity of a positive band to be a problem, since it is not known when a faint band should be considered truly positive. The criteria (band profiles) established by the manufacturer to confirm HTLV-1 and HTLV-2 infections in WB assays are excessively stringent and deserve a review. Taking into account the results obtained here, we suggest that samples presenting only one Gag band (p19 or p24) plus GD21 and rgp46-I or rgp-46-II should be considered HTLV-1 and HTLV-2 positive, respectively, since samples that demonstrated p24, GD21, and rgp46-I bands were confirmed to be HTLV-1 positive by the LIA and PCR. In contrast, we detected true HTLV-2 positivity in samples that showed p19, GD21, and rgp46-II bands. In addition, when Gag bands were undetectable but both envelope bands (GD21 and rgp46-I or rgp46-II) were present, it was impossible to rule out true HTLV-1 or HTLV-2 infection, since seroconversion could be taking place. Indeed, when all bands showed reactivity to HTLV-1 viral lysate antigens, even in the absence of rgp46-I, it was possible to confirm HTLV-1 infection.

In conclusion, the LIA was shown to be the best serological test for confirming HTLV-1 and HTLV-2 infections, regardless of whether individuals were HTLV monoinfected or coinfected. We further highlight the need to review some WB criteria based on our results and those reported previously by others. It remains to be determined whether the superior performance of the LIA was due to the less stringent criteria employed than for WB. Further studies are necessary to confirm these results in a variety of risk populations from Brazil and elsewhere.
